# Correlation between bone density, bone metabolism markers with lipid metabolism markers and body mass index

**DOI:** 10.1186/s12891-024-07284-6

**Published:** 2024-02-20

**Authors:** Hao Han, Ran Li, Dongming Fu, Hongyou Zhou, Zihao Zhan, Yi’ang Wu, Bin Meng

**Affiliations:** Department of Orthopedics, The First Affiliated Hospital of Soochow University, Suzhou, China

**Keywords:** Osteoporosis, Bone mineral density, Bone turnover markers, Lipid profile, Body mass index

## Abstract

**Purpose:**

We aimed to explore the relationship between bone mineral density (BMD), bone metabolism markers, and blood lipid-related indicators, body mass index (BMI) in elderly individuals.

**Methods:**

A retrospective analysis was conducted on 710 patients. Patients’ gender, age, height, weight, bone density values, T-scores, bone metabolism markers (including serum N-terminal propeptide of type I collagen (s-PINP), serum C-terminal telopeptide of type I collagen (s-CTX) and 1,25-dihydroxyvitamin D_3_ (1,25(OH)_2_D_3_) and lipid-related indicators (including total cholesterol (TC), low-density lipoprotein cholesterol (LDL-C), high-density lipoprotein cholesterol (HDL-C), triglycerides (TG) and Castelli index 1 (TC/HDL-C index) and Castelli index 2 (LDL-C/HDL-C index) were recorded. Correlations between variables were analyzed, and patients were grouped according to gender and T-score for intergroup comparisons.

**Results:**

HDL-C negatively correlates with BMD and s-CTX. TG, Castelli index, and BMI positively correlate with BMD. BMI negatively correlates with s-PINP. 1,25(OH)_2_D_3_ negatively correlates with TC, LDL-C, and Castelli index. LDL-C positively correlates with BMD in males, and TC negatively correlates with s-PINP. In females, HDL-C negatively correlates with BMD, and s-CTX positively correlates with Castelli index. 1,25(OH)_2_D_3_ negatively correlates with TC, LDL-C, and Castelli index. TG and Castelli index were higher in normal bone mass group, while HDL-C is higher in the osteoporosis group. TG and BMI positively predicted bone mass density, while HDL-C negatively predicted bone mass density.

**Conclusions:**

HDL-C may have a predictive role in osteoporosis, particularly in women. The likelihood of osteoporosis is lower in individuals with high BMI or hyperlipidemia. Some lipid metabolism markers can be used to predict osteoporosis, and further research is needed.

## Introduction

Osteoporosis is a systemic skeletal disorder characterized by reduced bone mass, deterioration of bone microarchitecture, increased bone fragility, and a consequent increase in fracture risk [1]. The most common clinical manifestations of osteoporosis are pain, spinal deformities, and fragility fractures [2], which significantly impact the quality of life of patients, earning it the moniker “the silent killer” [3]. In 2021, the United Nations General Assembly declared the decade of 2021 to 2030 as the “Decade of Healthy Ageing,” and identified musculoskeletal health as a key indicator of elderly health. On September 20, 2022, China’s National Health Commission highlighted the characteristics and situation of aging in China, predicting that by 2035, China would enter a phase of severe aging. The global burden of osteoporosis is a grave public health issue that is expected to worsen with the global trend towards an aging population. Specifically, deaths and disability-adjusted life years (DALYs) associated with low bone mineral density increased from 207,367 deaths and 8,588,936 DALYs in 1990 to 437,884 deaths and 16,647,466 DALYs in 2019, an increase of 111.16% and 93.82%, respectively [4]. In China, the overall prevalence of osteoporosis is 20.80%, with higher rates in females (23.57%, CI: 18.50–29.04) compared to males (12.22%, CI: 7.23–18.29), and a steady increase from 19.35% in 2001–05 to 21.30% in 2016–19. Prevalence rates rise with age for both sexes, from 10.22% in those aged 50–59 to 62.24% in those aged over 80. The prevalence rates for osteoporosis are considerably high among both Chinese men and women, particularly pronounced in elderly Chinese women. Therefore, with the rapid aging of the Chinese population, there is an urgent need for control measures and preventive management to address osteoporosis [5]. The most serious complication of osteoporosis is osteoporotic fractures. Data from the International Osteoporosis Foundation (IOF) indicate that fractures caused by osteoporosis are becoming an increasingly serious global issue. In China, the main osteoporotic fractures (including hip, vertebral, and wrist fractures) saw approximately 2.69 million new cases in 2015; this number is expected to rise to about 4.83 million by 2035 and about 5.99 million by 2050, with medical costs reaching up to 174.5 billion RMB [6].

Bone mineral density (BMD) is now recognized as directly associated to the incidence of osteoporotic fractures and is one of the main indicators for assessing the severity of osteoporosis. Bone turnover markers (BTMs) are metabolic products of bone tissue that can be used to reflect the activity of osteoblasts and the status of bone formation, such as serum procollagen type I N propeptide (s-PINP), and the activity of osteoclasts and the level of bone resorption, such as serum C-terminal telopeptide of type I collagen (s-CTX) [7]. According to Richard Eastell, BTMs are clinically applicable indicators that can be measured multiple times in individual patients, with high accuracy [8]. The active form of vitamin D, 1,25-dihydroxyvitamin D_3_ (1,25(OH)_2_D_3_), is a secosteroid hormone that regulates calcium and bone metabolism, controls cell proliferation and differentiation, and exerts immunoregulatory activities [9]. These indicators are very important in the study of osteoporosis.

Dual-energy X-ray absorptiometry (DXA) bone density testing is a commonly used and widely accepted method for assessing bone density, and is considered the gold standard for determining BMD due to its low radiation dose and high precision [10]. Measurements of bone turnover markers are typically used as adjunctive tools for diagnosing osteoporosis. However, not every patient undergoes DXA testing, usually only in elderly patients who have already experienced fractures or are suspected to have osteoporosis. Similarly, the measurement of bone turnover markers is not a routine clinical test. Therefore, it is crucial to identify a clinically routine test indicator and discover its correlation with bone density and bone metabolism.

Lipid metabolism, including total cholesterol (TC), low-density lipoprotein cholesterol (LDL-C), high-density lipoprotein cholesterol (HDL-C), and triglycerides (TG), can be assessed through routine biochemical tests. The Castelli index 1(TC/HDL-C index) and Castelli index 2(LDL-C/HDL-C index) is a marker of atherosclerosis [11] and is also included in the calculations. Body mass index (BMI) is an easily obtainable indicator that can be calculated by measuring a patient’s height and weight. Elderly populations tend to have a higher risk of developing both arteriosclerosis and osteoporosis. Consequently, elderly patients with osteoporosis have a heightened risk of cardiovascular diseases compared to those without osteoporosis [12]. Lipid-lowering medications, specifically statins, have been shown to maintain BMD and reduce the incidence of osteoporotic fractures [13]. Additionally, a high-fat diet has been observed to decrease BMD in animal models [14]. This suggests a definitive relationship between osteoporosis and lipid metabolism. We aim to establish a more precise correlation between BMD, bone turnover markers, lipid metabolism indices, and BMI. Such correlations would aid in enhancing the detection rate of early-stage osteoporosis, thereby facilitating early prevention and treatment strategies to reduce the incidence of osteoporotic fractures and improve the future quality of life for patients.

## Materials and methods

The objective of this study is to predict the incidence of osteoporosis through alterations in lipid metabolism. The overall prevalence rate of osteoporosis in China stands at 20.80%, with a permissible margin of error of 4%, a confidence level of 1-α = 0.95, and an allowance for a dropout-inflated sample size of 10%. Sample size calculations performed with PASS 2021 software determined that a minimum of 466 subjects is required for the investigation. We ultimately included a total of 702 patients who were admitted to the Department of Orthopedics at the First Affiliated Hospital of Soochow University between 2016 and 2021 for chronic neck, back, and leg pain, of which 548 were female and 154 were male. All included patients had no history of acute fractures, tumors, significant kidney or liver disease, or a history of taking medications that could affect lipid levels or bone mass. All female patients had no history of premature menopause, hysterectomy, or oophorectomy.

All patients underwent a comprehensive laboratory evaluation, with serum samples collected on the morning of the second day of hospitalization after overnight fasting. The evaluated parameters included lipid metabolism markers such as TC, LDL-C, HDL-C, and TG, as well as bone metabolism markers such as s-PINP, s-CTX and 1,25(OH)_2_D_3_. The Castelli index 1 and Castelli index 2 was calculated. Additionally, The BMI of each patient was also recorded, calculated as their weight in kilograms divided by the square of their height in meters. Inquire thoroughly about the patient’s history of smoking and diabetes. Upon discharge, record the total duration of their hospital stay.

These patients got an evaluation of their bone mass as part of the clinical evaluation. We measured the patient’s lumbar spine and femoral neck on both sides using dual energy X-ray absorptiometry, and we also got T-scores. According to World Health Organization standards, the results were divided into three groups: normal (T score from − 1.0 to 1.0 standard deviation), osteopenia (T score from − 1.0 to − 2.5 standard deviation), and osteoporosis (T score ≤ − 2.5 standard deviation) [15].

We used the Kolmogorove-Smirnov test to determine which variables were normally distributed. Student’s t-test and ANOVA were used for parametric tests. Student-Newman-Keuls test and Pearson correlation analysis were used to calculate intergroup differences. For non-parametric variables, Mann-Whitney’s U-test, Kruskall-Wallis test and Spearman’s correlation analysis were used. Multiple linear regression analysis is used to analyze the relationship between multiple independent variables and dependent variables. Utilize AUC (Area Under the Curve) analysis to evaluate the accuracy and reliability of prediction outcomes.

## Results

The average age of the patients involved in the study was 68.42 ± 9.10 years (ranging from 45 to 97 years), with an average BMI of 23.57 ± 3.46 kg/m^2^, and an average hospital stay of 10.46 ± 2.90 days. The average BMD values for lumbar vertebrae (L1–L4), left femoral neck, and right femoral neck were 0.77 ± 0.18 g/cm^2^, 0.76 ± 0.15 g/cm^2^, and 0.75 ± 0.14 g/cm^2^, respectively. The bone turnover markers s-CTX and s-PINP had median values of 0.93 (interquartile range [IQR]: 0.52, 1.22) µmol/L and 64.50 (IQR: 38.18, 78.64) µmol/L, respectively. The median level of 1,25(OH)_2_D_3_ was 19.10 (IQR: 13.50, 23.60) µmol/L. The lipid profile showed median TC of 4.80 (IQR: 4.09, 5.36) µmol/l, TG of 1.42 (IQR: 0.94, 1.72) µmol/l, LDL-C of 2.83 (IQR: 2.21, 3.37) µmol/l, and HDL-C of 1.21 (IQR: 0.97, 1.38) µmol/l. The average Castelli index 1 was 4.18 (IQR: 3.33, 4.89), and the average Castelli index 2 was 2.49 (IQR: 1.82, 3.10).

Table 1 presents the biological indicator information of the samples grouped by gender. It is evident that these indicators show certain differences between males and females. Therefore, it is essential to conduct discussions both for the overall sample and separately based on gender.

**Table 1:** Lipid profile, bone parameters and some biological variables of the patients

	Men (*n* = 154)	Women (*n* = 548)	Significance
Total Cholesterol (µmol/L)	4.34(3.81,5.06)	4.82(4.17,5.43)	Z=-4.58, *P*<0.001
LDL-C (µmol/L)	2.62(2.15,3.22)	2.78(2.23,3.45)	Z=-1.95, NS
HDL-C (µmol/L)	1.06(0.90,1.30)	1.17(1.00,1.40)	Z=-1.95, *P* = 0.001
Triglycerides (µmol/L)	1.15(0.90,1.51)	1.30(0.94,1.76)	Z=-2.56, *P* = 0.01
Castelli index 1	4.04(3.21,4.81)	4.02(3.36,4.90)	Z=-0.479, NS
Castelli index 2	2.41(1.79,3.14)	2.36(1.83,3.07)	Z=-0.673, NS
s-CTX (µmol/L)	0.75(0.52,0.99)	0.83(0.52,1.26)	Z=-2.05, *P* = 0.04
s-PINP (µmol/L)	49.05(36.02,69.24)	60.09(39.57,81.64)	Z=-3.14, *P* = 0.002
1,25(OH)_2_D_3_ (µmol/L)	18.45(13.78,23.38)	17.80(13.40,23.68)	Z=-0.721, NS
Left femoral neck BMD (g/cm^2^)	0.86 ± 0.15	0.73 ± 0.13	t = 10.53, *P*<0.001
Right femoral neck BMD (g/cm^2^)	0.85 ± 0.14	0.73 ± 0.13	t = 10.39, *P*<0.001
Lumbar spine BMD (g/cm^2^)	0.92 ± 0.19	0.73 ± 0.15	t = 11.06, *P*<0.001
BMI (kg/m^2^)	23.45 ± 3.20	23.60 ± 3.53	t=-0.474, NS
Age (years)	68.10 ± 9.69	68.51 ± 8.92	t=-0.492, NS
Duration of hospitalization (days)	10.50 ± 2.98	10.45 ± 2.87	t = 0.173, NS
Diabetes history (yes/no)	57/97	193/355	χ2 = 0.169, NS
Smoking history(yes/no)	70/84	90/458	χ2 = 57.58, *P*<0.001

NS = *p*>0.05

In general, total cholesterol and LDL-C are negatively correlated with1,25(OH)_2_D_3_ levels. HDL-C is significantly negatively correlated with lumbar spine BMD, bilateral femoral neck BMD, and the bone resorption marker s-CTX. Simultaneously, triglycerides, Castelli index 1, Castelli index 2, and BMI are positively correlated with lumbar spine and bilateral femoral neck BMD. Castelli index 1 and Castelli index 2 are negatively correlated with serum 1,25(OH)_2_D_3_ levels. Castelli index 2 is positively correlated with s-CTX. BMI is negatively correlated with s-PINP (Table 2).

**Table 2:** Correlations between lipid profile, BMI, BMD and BTMs

	Lumbar spine BMD (g/cm^2^)	Left femoral neck BMD (g/cm^2^)	Right femoral neck BMD (g/cm^2^)	s-CTX (µmol/L)	s-PINP (µmol/L)	1,25(OH)_2_D_3_ (µmol/L)
Total cholesterol (µmol/L)	ρ=-0.025 NS	ρ=-0.007 NS	ρ = 0.018 NS	ρ=-0.016 NS	ρ=-0.036 NS	ρ=-0.105 *p* = 0.005
LDL-C (µmol/L)	ρ = 0.004 NS	ρ = 0.029 NS	ρ = 0.049 NS	ρ = 0.034 NS	ρ = 0.014 NS	ρ=-0.120 *p* = 0.001
HDL-C (µmol/L)	ρ=-0.157 *p*<0.001	ρ=-0.163 *p*<0.001	ρ=-0.154 *p*<0.001	ρ=-0.076 *p* = 0.045	ρ=-0.066 NS	ρ = 0.033 NS
Triglyceride (µmol/L)	ρ = 0.221 *p*<0.001	ρ = 0.217 *p*<0.001	ρ = 0.217 *p*<0.001	ρ=-0.037 NS	ρ=-0.028 NS	ρ=-0.050 NS
Castelli index 1	ρ = 0.138 *p*<0.001	ρ = 0.170 *p*<0.001	ρ = 0.175 *p*<0.001	ρ = 0.058 NS	ρ = 0.028 NS	ρ=-0.109 *p* = 0.004
Castelli index 2	ρ = 0.111 *p*<0.001	ρ = 0.149 *p*<0.001	ρ = 0.151 *p*<0.001	ρ = 0.075 *p* = 0.047	ρ = 0.051 NS	ρ=-0.112 *p* = 0.003
BMI (kg/m^2^)	ρ = 0.331 *p*<0.001	ρ = 0.441 *p*<0.001	ρ = 0.439 *p*<0.001	ρ=-0.065 NS	ρ = 0.-087 *p* = 0.022	ρ = 0.014 NS

When examining male and female patients separately, LDL-C is correlated with bilateral femoral neck BMD in male patients, but not with lumbar spine BMD in male patients. It is not correlated with lumbar spine and bilateral femoral neck BMD in female patients. In females, HDL-C is negatively correlated with lumbar spine and bilateral femoral neck BMD, while this correlation is not significant in male patients. Regardless of gender, triglycerides, Castelli index 1, Castelli index 2, and BMI are all positively correlated with lumbar spine and bilateral femoral neck BMD (Table 3).

**Table 3:** Analysis of men and women separately: correlations between lipid profile and BMD

	Lumbar spine BMD (g/cm^2^)		Left femoral neck BMD (g/cm^2^)		Right femoral neck BMD (g/cm^2^)	
	Men	Women	Men	Women	Men	Women
Total cholesterol (µmol/L)	ρ = 0.139 NS	ρ = 0.024 NS	ρ = 0.118 NS	ρ = 0.019 NS	ρ = 0.118 NS	ρ = 0.053 NS
LDL-C (µmol/L)	ρ = 0.133 NS	ρ = 0.009 NS	ρ = 0.207 *p* = 0.010	ρ = 0.014 NS	ρ = 0.206 *p* = 0.010	ρ = 0.037 NS
HDL-C (µmol/L)	ρ=-0.088 NS	ρ=-0.127 *p* = 0.003	ρ=-0.114 NS	ρ=-0.130 *p* = 0.002	ρ=-0.117 NS	ρ=-0.118 *p* = 0.006
Triglycerides (µmol/L)	ρ = 0.334 *p*<0.001	ρ = 0.279 *p*<0.001	ρ = 0.364 *p*<0.001	ρ = 0.244 *p*<0.001	ρ = 0.361 *p*<0.001	ρ = 0.225 *p*<0.001
Castelli index 1	ρ = 0.215 *p* = 0.008	ρ = 0.143 *p* = 0.001	ρ = 0.261 *p* = 0.001	ρ = 0.158 *p*<0.001	ρ = 0.227 *p* = 0.005	ρ = 0.165 *p*<0.001
Castelli index 2	ρ = 0.171 *p* = 0.034	ρ = 0.092 *p* = 0.031	ρ = 0.270 *p* = 0.001	ρ = 0.115 *p* = 0.007	ρ = 0.271 *p* = 0.001	ρ = 0.117 *p* = 0.006
BMI (kg/m^2^)	ρ = 0.464 *p*<0.001	ρ = 0.364 *p*<0.001	ρ = 0.584 *p*<0.001	ρ = 0.462 *p*<0.001	ρ = 0.571 *p*<0.001	ρ = 0.456 *p*<0.001

In male patients, only s-PINP is negatively correlated with TC, while in female patients, s-CTX is negatively correlated with HDL-C, s-PINP is negatively correlated with BMI, and positively correlated with Castelli index 1 and Castelli index 2. Additionally, 1,25(OH)_2_D_3_ is negatively correlated with TC, LDL-C, Castelli index 1, and Castelli index 2 (Table 4).

**Table 4:** Analysis of men and women separately: correlations between lipid profile and BTMs

	s-CTX (µmol/L)		s-PINP (µmol/L)		1,25(OH)_2_D_3_ (µmol/L)	
	Men	Women	Men	Women	Men	Women
Total cholesterol (µmol/L)	ρ=-0.034 NS	ρ=-0.027 NS	ρ=-0.213 *p* = 0.008	ρ=-0.016 NS	ρ=-0.11 NS	ρ=-0.097 *p* = 0.023
Triglycerides (µmol/L)	ρ = 0.010 NS	ρ=-0.057 NS	ρ=-0.137 NS	ρ=-0.017 NS	ρ=-0.056 NS	ρ=-0.040 NS
HDL-C (µmol/L)	ρ=-0.008 NS	ρ=-0.109 *p* = 0.011	ρ=-0.085 NS	ρ=-0.074 NS	ρ = 0.028 NS	ρ = 0.039 NS
LDL-C (µmol/L)	ρ = 0.043 NS	ρ = 0.043 NS	ρ=-0.135 NS	ρ = 0.038 NS	ρ=-0.101 NS	ρ=-0.123 *p* = 0.004
Castelli index 1	ρ = 0.082 NS	ρ = 0.090 *p* = 0.036	ρ=-0.084 NS	ρ = 0.052 NS	ρ=-0.077 NS	ρ=-0.115 *p* = 0.007
Castelli index 2	ρ=-0.071 NS	ρ = 0.115 *p* = 0.007	ρ=-0.040 NS	ρ = 0.079 NS	ρ=-0.061 NS	ρ=-0.126 *p* = 0.003
BMI (kg/^2^)	ρ=-0.068 NS	ρ=-0.067 NS	ρ=-0.021 NS	ρ=-0.104 *p* = 0.015	ρ = 0.083 NS	ρ=-0.004 NS

**Fig. 1:**
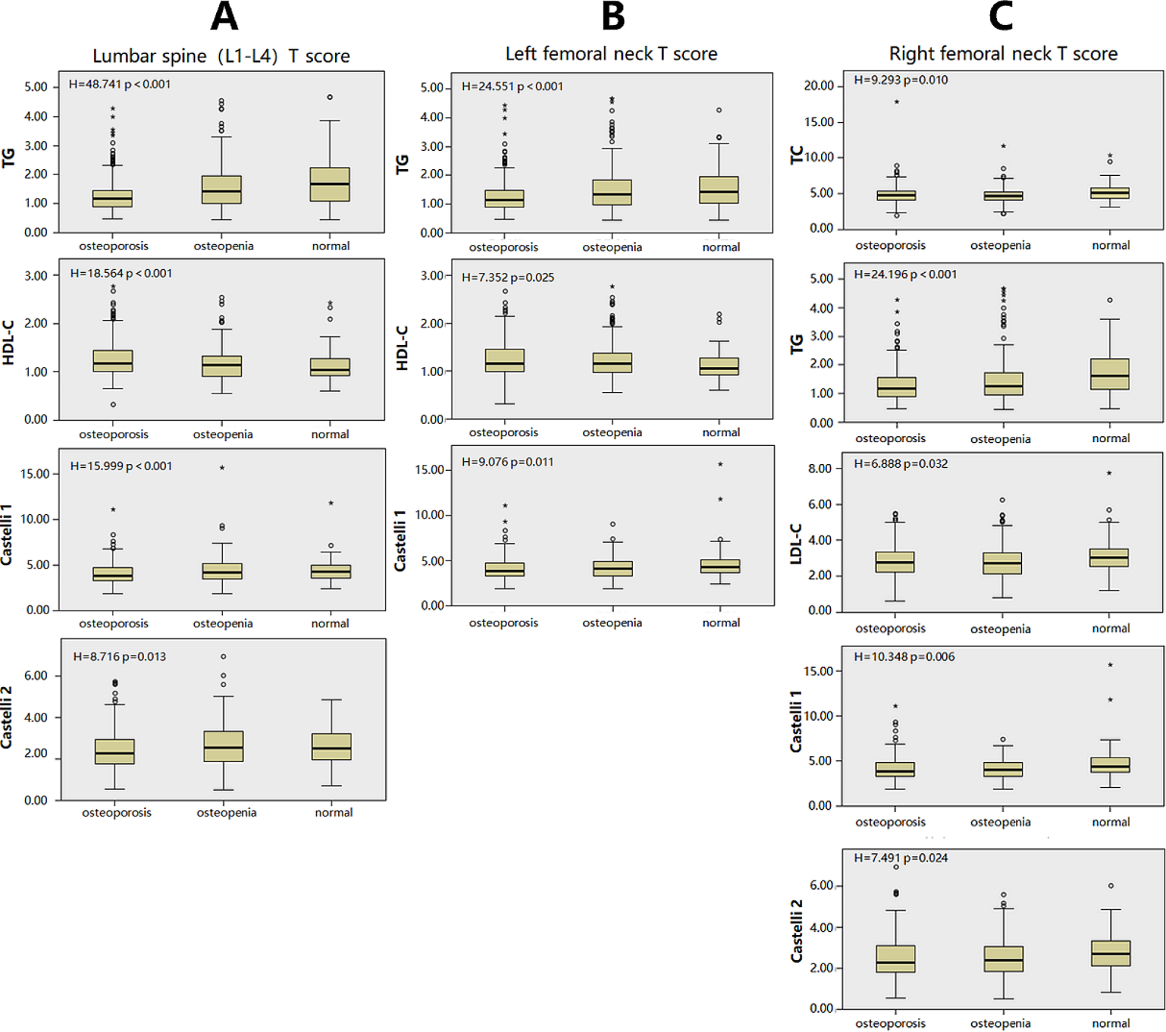
Patients were divided into normal, Osteopenia and Osteoporosis according to T score: **A**. according to Lumbar spine (L1-L4) T score; **B**. according to Left femoral neck T score; C. according to Right femoral neck T score

When we divided the population into groups based on lumbar spine T-scores, namely the normal bone mass group, osteopenia group, and osteoporosis group, we observed significant between-group differences in TG (H = 48.741, *p* < 0.001), HDL-C (H = 18.564, *p* < 0.001), Castelli index 1 (H = 15.999, *p* < 0.001), and Castelli index 2 (F = 8.716, *p* = 0.013). TG, Castelli index 1, and Castelli index 2 were higher in the normal bone mass group, while HDL-C was higher in the osteoporosis group. Similarly, when we investigated the groups based on the T-scores of the left femoral neck, we found significant between-group differences in TG (H = 24.551, *p* < 0.001), HDL-C (H = 7.352, *p* = 0.025), and Castelli index 1 (H = 9.076, *p* = 0.011). TG and Castelli index 1 were higher in the normal bone mass group, while HDL-C was higher in the osteoporosis group. Furthermore, when studying the groups based on the T-scores of the right femoral neck, we observed significant between-group differences in TC (H = 9.293, *p* = 0.010), TG (H = 24.196, *p* < 0.001), LDL-C (H = 6.888, *p* = 0.032), Castelli index 1 (H = 10.348, *p* = 0.006), and Castelli index 2 (H = 7.491, *p* = 0.024), all of which were higher in the normal bone mass group (Table 5; Fig. 1).

**Table 5:** Lipid variables among patients with normal 、osteopenia or osteoporosis T-score

		Lumbar spine (L1-L4) T score			Left femoral neck T score			Right femoral neck T score		
		Normal	Osteopenia	Osteoporosis	Normal	Osteopenia	Osteoporosis	Normal	Osteopenia	Osteoporosis
Total cholesterol (µmol/L)	Median	4.76(3.86,5.46)	4.74(4.11,5.33)	4.71(4.14,5.39)	4.67(4.08,5.51)	4.73(4.07,5.34)	4.71(4.13,5.37)	5.07(4.31,5.76)	4.64(4.06,5.29)	4.74(4.06,5.40)
	H	0.625			0.196			9.293		
	P	NS			NS			0.010		
Triglycerides (µmol/L)	Median	1.67(1.08,2.24)	1.43(1.00,1.94)	1.16(0.88,1.46)	1.42(1.03,1.95)	1.33(0.98,1.86)	1.15(0.87,1.47)	1.61(1.15,2.21)	1.26(0.94,1.73)	1.17(0.89,1.56)
	H	48.741			24.551			24.196		
	P	<0.001			<0.001			<0.001		
HDL-C (µmol/L)	Median	1.04(0.91,1.28)	1.14(0.90,1.32)	1.18(1.01,1.44)	1.06(0.92,1.28)	1.15(0.97,1.37)	1.16(0.99,1.48)	1.12(0.94,1.35)	1.14(0.97,1.36)	1.16(0.99,1.44)
	H	18.564			7.352			2.567		
	P	<0.001			0.025			NS		
LDL-C (µmol/L)	Median	2.75(2.06,3.36)	2.83(2.22,3.39)	2.75(2.22,3.37)	2.90(2.22,3.54)	2.73(2.20,3.33)	2.78(2.22,3.37)	3.03(2.51,3.54)	2.71(2.16,3.30)	2.75(2.22,3.37)
	H	0.501			0.769			6.888		
	P	NS			NS			0.032		
Castelli index 1	Median	4.25(3.59,5.00)	4.16(3.44,5.17)	3.84(3.27,4.69)	4.27(3.67,5.12)	4.10(3.31,4.89)	3.85(3.27,4.73)	4.33(3.72,5.40)	4.00(3.31,4.82)	3.86(2.27,4.84)
	H	15.999			9.076			10.348		
	P	<0.001			0.011			0.006		
Castelli index 2	Median	2.50(1.94,3.22)	2.53(1.87,3.33)	2.27(1.76,2.94)	2.63(2.01,3.31)	2.40(1.84,3.07)	2.25(1.77,3.08)	2.69(2.12,3.34)	2.37(1.82,3.05)	2.28(1.78,3.10)
	H	8.716			5.985			7.491		
	P	0.013			NS			0.024		

Multiple linear regression analysis was conducted to assess the predictive ability of various lipid metabolism indicators on bone density. For lumbar spine bone density regression equation, the results were significant with F = 24.027, *P* < 0.001. In this equation, TG (β = 0.178, *P* < 0.001) and BMI (β = 0.304, *P* = 0.009) positively predicted lumbar spine bone density, while TC, HDL-C, and LDL-C were not significant predictors of lumbar spine bone density. For left femoral neck bone density regression equation, the results were significant with F = 37.799, *P* < 0.001. In this equation, TG (β = 0.115, *P* = 0.002) and BMI (β = 0.405, *P* < 0.001) positively predicted left femoral neck bone density, while HDL-C (β=-0.103, *P* = 0.009) negatively predicted left femoral neck bone density. TC and LDL-C were not significant predictors of left femoral neck bone density. For right femoral neck bone density regression equation, the results were significant with F = 36.984, *P* < 0.001. In this equation, TG (β = 0.107, *P* = 0.003) and BMI (β = 0.402, *P* < 0.001) positively predicted right femoral neck bone density, while HDL-C (β=-0.103, *P* = 0.009) negatively predicted right femoral neck bone density. TC and LDL-C were not significant predictors of right femoral neck bone density (Table 6).

**Table 6:** Multiple linear regression analysis was performed to evaluate the factors associated with changes in lumbar spine T-score and femoral neck T-score

	Lumbar spine (L1-L4) T score					Left femoral neck T score					Right femoral neck T score				
	β	t	p	F	P	β	t	p	F	P	β	t	p	F	P
TC	-0.065	-1.178	0.239	24.027	<0.001	0.005	0.087	0.931	37.799	<0.001	0.018	0.346	0.730	36.984	<0.001
TG	0.178	4.712	<0.001			0.115	3.183	0.002			0.107	2.936	0.003		
HDL-C	-0.044	-1.065	0.287			-0.103	-2.620	0.009			-0.107	-2.712	0.007		
LDL-C	0.006	0.123	0.902			0.015	0.303	0.762			0.021	0.441	0.659		
BMI	0.304	8.591	<0.001			0.405	11.926	<0.001			0.402	11.820	<0.001		

We conducted an AUC analysis to establish the threshold values for HDL-C. The results indicated that predicting lumbar spine BMD using HDL-C is feasible to a certain extent, although its discriminative ability is not high, with an AUC of 0.590. Our analysis suggests that an HDL-C value higher than 1.365 can predict a decrease in lumbar spine BMD, with a sensitivity of 0.330 and a specificity of 0.810 (Table 7).

**Table 7:** AUC Analysis for Predicting Bone Density at Various Sites Using HDL-C

Predictive Indicator	Accuracy of discrimination [AUC (95% CI)]	Default error	P value	Sensibility	Specificity	Suggested cutoffs
Lumbar spine BMD	0.590(0.548, 0.632)	0.021	<0.001	0.330	0.810	1.365
Left femoral neck BMD	0.542(0.498, 0.585)	0.022	0.062	-	-	-
Right femoral neck BMD	0.533(0.489, 0.577)	0.023	0.138	-	-	-

## Discussion

This study investigated the correlations among lipid metabolism markers, BMI, BMD, and BTMs. The findings suggest that individuals with elevated levels of HDL-C generally exhibit lower BMD. Conversely, subjects with high triglyceride levels and those with a higher BMI tend to have a reduced likelihood of developing osteoporosis. Pertaining to markers of bone turnover, s-CTX demonstrated a negative correlation with HDL-C in the general population, with this association being more significant in female cohorts. In male subjects, there was a negative correlation identified between total cholesterol levels and the bone formation marker s-PINP.

HDL-C and Castelli index are commonly used to assess cardiovascular disease risk. High levels of high-density lipoprotein cholesterol and lower Castelli index are typically considered to be a healthier state and indicate a lower risk of cardiovascular disease [16]. However, in our study involving a cohort of 710 patients with an average age of 68, we observed a negative correlation between HDL-C and BMD, while Castelli index were positively correlated with BMD. When we categorized patients into three groups (Normal, Osteopenia, and Osteoporosis) based on T-scores of the lumbar spine and left femoral neck, we observed that HDL-C levels were higher in the Osteoporosis group, while the Castelli index was higher in the Normal group. This finding is highly similar to that of Jinyoung Kim’s study, which investigated 4,323 participants in Korea (2,286 men and 2,037 women) and found a negative correlation between HDL-C and BMD in the general population, although the correlation was not significant when analyzed separately by gender [17]. Rongtao Cui’s study on 1,035 men and 3,953 women healthy volunteers found that individuals with HDL-C levels ≥ 1.56 mmol/L had a higher incidence of osteoporosis [18]. This may be because oxysterol play an important role in osteogenic differentiation, and HDL-C can remove oxysterol from the peripheral circulation. As Kha et al. mentioned, in this mechanism, a high level of HDL-C would inhibit osteogenic differentiation [19].

In the overall analysis, we did not observe a significant correlation between LDL-C and bone density. However, when we conducted separate analyses based on gender, we found a positive correlation between LDL-C and BMD in male patients, particularly in both sides of the femoral neck. On the other hand, in female patients, we observed a negative correlation between HDL-C levels and BMD, both in the lumbar spine and both sides of the femoral neck. These gender-specific differences in the correlation between lipid levels and bone density may be influenced by various factors, including genetics, lifestyle, nutrition, and hormonal levels. Additionally, the smaller sample size of male patients in our study might have contributed to the observed differences. Further research is needed to better understand the underlying mechanisms and factors contributing to these associations. Furthermore, based on the results of multiple regression analysis, it has been found that HDL-C can negatively predict the bone density of the bilateral hips, with no statistically significant association with lumbar spine bone density. This may be attributed to the fact that the hip region is primarily composed of cortical bone, whereas the lumbar spine consists of a greater proportion of trabecular bone [20]. HDL-C may exert differing effects on these two distinct types of bone tissue, resulting in variations in the relationship with bone density at different anatomical sites.

The results of the AUC analysis suggest that it is somewhat feasible to predict lumbar spine BMD with HDL-C levels. An HDL-C value higher than 1.365 can predict a decrease in lumbar spine BMD, with a sensitivity of 0.330 and a specificity of 0.810. On one hand, HDL-C can predict osteoporosis in the lumbar spine to a certain extent, but it may result in a high false-negative rate. Therefore, it is essential to combine this with other diagnostic methods for a comprehensive diagnosis. However, given the high specificity of HDL-C in predicting osteoporosis, it is recommended that a bone density test be conducted to clarify the bone mass situation when elevated HDL-C levels (> 1.365 µmol/L) are observed.

We have also found a positive correlation between triglycerides and parameters related to bone mass. When we grouped the patients based on their T-scores, we also observed that TG levels were higher in the normal bone density group. This may be because triglycerides can provide energy after being broken down in the human body, which helps maintain normal bone metabolism. The impact of the lipid profile on BMD can be elucidated through multiple biological mechanisms. Initially, the nuclear hormone receptor peroxisome proliferator-activated receptor gamma (PPARγ) may play a role in mediating the relationship between lipid biomarkers and BMD. PPARγ is activatable by lipid metabolites. Elevated PPARγ levels result in the suppression of osteoblastic activity, leading to an increase in bone resorption [21]. Higher levels of lipids are associated with an increase in oxidized lipids and a higher level of oxidative stress. Increased oxidative stress can inhibit the differentiation of osteoblasts and promote the differentiation of adipocytes [22, 23]. Moreover, higher serum TG levels are positively correlated with increased bone marrow fat, which leads to a decrease in trabecular bone density [24, 25]. A study conducted by Rucha Saoji on 293 women from northeastern India showed a positive correlation between triglycerides and BMD, which is an important predictor of osteopenia and osteoporosis [26].

Several other authors have also analyzed the relationship between lipid profiles and bone alterations in different populations or in patients with specific diseases and have come to different conclusions. Ismail Alay’s study of 452 postmenopausal women showed that only lumbar bone mineral density showed a negative correlation with LDL-C, while no other lipid markers were significantly correlated with bone mineral density [27]. But when we grouped the patients based on their T scores of the right hip’s femoral neck, we found that LDL-C levels were higher in the normal bone density group. Po-Yin Chang’s study of 2,062 premenopausal or early perimenopausal women with no history of fracture found that increased triglyceride levels may increase the probability of fracture [28]. In a study by Irene Zolfaroli et al. in a total of 667 of the 1304 screened women, HDL-C was found to be positively correlated with bone mineral density in the lumbar spine and femoral neck, while the other indices, including TC, TG, and LDL-C, were not significantly correlated with bone mineral density [29]. Based on our analysis, we believe that TC levels may be higher in patients with normal bone density compared to those with lower bone density. The explanation for these differences is not clear, but the reasons for this could be attributed to differences in study design, population selection, as well as the influence of different races, ethnicities, genetic backgrounds, and lifestyles on serum lipid profiles and bone health.

Our study also revealed a correlation between bone turnover markers and lipid metabolism indicators. S-CTX is a serum marker used to assess the degree of bone remodeling. When bone tissue is being broken down, the C-terminal telopeptide of type I collagen is released into the bloodstream, resulting in the formation of s-CTX. Therefore, high levels of s-CTX usually indicate increased bone remodeling activity, with bone tissue undergoing continuous breakdown and reconstruction. On the other hand, s-PINP is another serum marker used to evaluate the formation of new bone tissue during bone remodeling. The N-terminal propeptide of type I procollagen is a marker of newly formed bone tissue and is released into the bloodstream during the early stages of bone remodeling. Consequently, high levels of s-PINP typically suggest an increase in the formation of new bone tissue, indicating that bone remodeling and strengthening are occurring. We found a negative correlation between s-CTX and HDL-C in the general population. However, when we analyzed the population by gender, s-CTX only showed a negative correlation with HDL-C in females, while there was no significant correlation in males. Meanwhile, in men, there was a negative correlation between total cholesterol and the bone formation marker s-PINP. These phenomena may be caused by the differences in hormone levels between females and males. The research findings also indicate that there is a positive correlation between Castelli index 2 and s-CTX. There are also studies suggesting that HDL-C can induce apoptosis of osteoclasts, thereby reducing the levels of bone resorption-related factors. Huang et al. found that HDL-C promotes osteoclast cholesterol efflux by upregulating ABCG1 expression, which disrupts cholesterol homeostasis in osteoclasts and consequently induces osteoclast apoptosis and affects its formation [30]. This effect may coexist with the previously mentioned high-level HDL-C inhibition of bone differentiation, and it may manifest different results in different genders. In this study, it was found that there is a positive correlation between s-PINP and the Castelli index in females, while this correlation was not significant in male patients. Although numerous studies have indicated that HDL-C impacts osteoblastic differentiation and osteoclast apoptosis, the correlation between HDL-C and BTMs was not particularly significant in our study. HDL-C plays multifaceted roles in many other biological processes, including inflammation, oxidative stress, nitric oxide production, and plasma glucose homeostasis regulation. As previously mentioned, oxysterols play a crucial role in osteoblastic differentiation, and HDL-C can clear oxysterols from the peripheral circulation. Furthermore, Brodeur and colleagues have demonstrated that oxidized LDL-C can induce osteoblast apoptosis, an effect that can be mitigated by the addition of HDL-C [31]. In osteoclasts, HDL-C particles remove cholesterol, thereby inducing apoptosis, while cholesterol delivery via LDL-C can enhance osteoclast survival [32]. Therefore, I believe the reason for this contradiction may be twofold: on one hand, the effect of HDL-C on BTMs might be multifaceted, leading to a net effect that does not significantly alter BTMs levels as reflected in the data. On the other hand, HDL-C may not affect bone mass by directly promoting the synthesis of collagen by osteoblasts or by activating osteoclastic bone resorption. Hence, HDL-C appears to have no significant correlation with BTMs. Currently, research on the correlation between HDL-C and BMD or BTMs has not reached a consensus. There seems to be a relationship, but it is highly context-specific, and existing data are insufficient to determine the specifics of this relationship. More extensive sample sizes and additional laboratory studies may be required to further clarify their relationship.

The results also showed that 1,25(OH)_2_D_3_ was negatively correlated with total cholesterol and low-density lipoprotein cholesterol, and correspondingly, the Castelli index were negatively correlated with 1,25(OH)_2_D_3_. When we conducted a gender-specific analysis, we found that this negative correlation only appeared in women, and no significant correlation was found in men, which may be due to the smaller sample size of men. Some studies have shown that individuals with elevated vitamin D levels tend to have lower levels of LDL-C [33]. Li-ming Tan’s study on 291 patients found that decreased 1,25(OH)_2_D_3_ levels increase the risk of osteoporosis. Therefore, when patients present with hypercholesterolemia or elevated LDL-C levels, the possibility of vitamin D deficiency should be considered, and efforts should be made to supplement vitamin D to prevent the development of osteoporosis.

In our research, BMI showed a significant positive correlation with bone density, and this correlation still existed when the population was studied separately by gender. Similar conclusions have been reported in many studies. The results of L Jia et al.‘s analysis of 128 postmenopausal women with osteoporotic fractures suggested that the smaller the BMI value, the greater the BMD loss [34]. After studying 900 elderly patients, Asuman Doğan concluded that overweight individuals have significantly higher BMD levels compared to those with normal weight [35]. However, this is not a universally accepted conclusion. For example, Ming Ma et al. conducted a cross-sectional study using data from the National Health and Nutrition Examination Survey from 2005 to 2006, 2007–2008, 2009–2010, 2013–2014, and 2017–2018. The study included 10,910 participants, and the conclusion was that the relationship between BMI and BMD is not a simple linear relationship, and there is a saturation point. Maintaining a slightly overweight BMI value can achieve optimal BMD [36]. A Auslander et al. studied the correlation between BMI and BMD in young, sedentary women and found that BMI cannot predict BMD [37]. In our study, we also found a negative correlation between BMI and the bone formation marker s-PINP, and this relationship was more significant in females. This suggests that although individuals with high BMI may have increased BMD, their bone formation capacity may actually be weakened, which is not beneficial for bone health. Therefore, individuals with low BMI should take early preventive measures against osteoporosis, and those with high BMI should not assume that they are not at risk for osteoporosis, and should pay even greater attention to protecting their bones.

The multiple linear regression analysis revealed that TG and BMI positively predicted lumbar spine and bilateral femoral neck bone density, which aligns with the findings from previous results. However, HDL-C showed a negative association with bilateral femoral neck bone density, but it was not predictive of lumbar spine bone density. This discrepancy may be attributed to differences in bone structure and function between the two anatomical locations. The study conducted by Peng Niu et al. with 440 participants also reported a negative correlation between blood HDL-C levels and lunar total femur and femoral neck bone density. Importantly, this negative correlation persisted even after adjusting for various covariates such as age, sex, smoking, alcohol consumption, exercise status, history of heart disease, and hypertension [38]. The observed relationships between lipid parameters and bone density suggest that lipid metabolism may play a role in bone health and remodeling. The differing associations between HDL-C and bone density at different anatomical sites may indicate site-specific effects of lipid metabolism on bone metabolism. Further research is warranted to better understand the underlying mechanisms linking lipid metabolism to bone health and to explore the implications of these associations in the context of bone-related diseases and overall health outcomes.

Our study has several limitations that warrant acknowledgment. Firstly, both bone and lipid metabolism are subject to the influence of a myriad of factors, with some unknown confounders possibly remaining unaccounted for. Secondly, lipid levels are dynamically affected by the physiological state of the body; we have only measured the blood lipid levels of participants on a single occasion, which may introduce potential deviations from their true lipid profiles. Longitudinal studies incorporating multiple measurements over time would be more appropriate for assessing the temporal relationships between lipid metabolism and bone health. Additionally, our research was concentrated on a specific demographic, all being East Asian Chinese, and ethnic variability may influence the outcomes, limiting the generalizability of our results to other populations or ethnic groups. Furthermore, the sample size of male participants was comparatively small, which could have affected the statistical power and might account for some findings that were not statistically significant.

## Conclusion

Broadly speaking, lipid metabolism-related indicators and BMI are intimately linked with bone density and bone metabolism markers, and they are important predictive markers for osteoporosis. For patients visiting non-orthopedic outpatient clinics, the likelihood of undergoing bone density or bone metabolism testing is not high. However, these patients are more likely to undergo lipid profile tests, which are also standard in health check-ups. Therefore, we hope to predict the likelihood of these patients developing osteoporosis through the results of tests they are more likely to undergo. For instance, clinicians should be vigilant about individuals with elevated HDL-C levels, particularly postmenopausal women, who may be at risk of reduced bone mass. Additionally, individuals with a lower BMI have a higher risk of osteoporosis. For these patients, close monitoring of BMD and early intervention may be necessary to reduce complications such as osteoporotic fractures, thereby enhancing the patient’s quality of life.

## Data Availability

The data that support the findings of this study are included in this manuscript, and the original files are available from the corresponding author upon reasonable request.、.

## Abbreviations

LDL-C: Low density lipoprotein cholesterol
HDL-C: High density lipoprotein cholesterol
TC: Total cholesterol
TG: Triglycerides
s-CTX: Serum C-terminal telopeptide of type I collagen
s-PINP: Serum N-terminal propeptide of type I collagen
1,25-dihydroxyvitamin D_3_: 1,25(OH)_2_D_3_
BMD: Bone mineral density
BMI: Body mass index
NS: No significance
AUC: Area Under Curve
PPARγ: Peroxisome proliferator-activated receptor gamma
IQR: Interquartile range
